# Predictors of the Number of Installs in Psychiatry Smartphone Apps: Systematic Search on App Stores and Content Analysis

**DOI:** 10.2196/15064

**Published:** 2019-11-19

**Authors:** Mariana Pinheiro, Madalena Serra, Nuno Pereira-Azevedo

**Affiliations:** 1 Psychiatrical Service of the Psychiatry and Mental Health Department Centro Hospitalar do Tâmega e Sousa Guilhufe Portugal; 2 Department of Psychiatry and Mental Health Hospital do Espírito Santo Évora Portugal; 3 Department of Urology Centro Hospitalar de Entre o Douro e Vouga Santa Maria da Feira Portugal

**Keywords:** eHealth, mHealth, mobile applications, psychiatry

## Abstract

**Background:**

Mental health is integral to our salubrity, but mental disorders are very debilitating and common. Therefore, it is critical to provide accessible, timely, and inexpensive mental care. This can be done through mobile health (mHealth), namely, mobile medical apps, which are gaining popularity among clinicians and patients. mHealth is a fast-paced field, and there is significant variation in the number of installs among psychiatry apps. However, the factors that influence psychiatry app installs have yet to be studied.

**Objective:**

This study aimed to identify predictors of the number of app installs in psychiatry.

**Methods:**

A literature review identified which factors influence app installs. Psychiatry apps available in the Google Play Store were reviewed, and publicly available data were collected. A multivariate ordinal logistic regression analysis was performed to evaluate the effect of said factors on the number of installs.

**Results:**

Our search identified 128 psychiatry apps: 2.3% (3/128) had never been installed, approximately half (53.1%, 68/128) had less than 500 installs, and only 0.8% (1/128) had over 10,000,000 installs. A multivariate logistic regression analysis identified that apps with a lower price (*P*<.001), a higher rating (*P*<.001), optional in-app purchases (*P*<.001), and age restriction (*P*=.04) had a higher number of installs. The involvement of a psychiatrist or other health care professional (HCP) had no statistically significant influence on the number of installs. Only data from the Google Play Store and the developers’ websites were available for analysis, and the depth of involvement of HCPs was impossible to document.

**Conclusions:**

Psychiatry apps with a lower price, optional in-app purchases, age restriction, and a higher rating are expected to have a higher number of installs. Unlike other medical fields, in this study, the explicit participation of psychiatrists in app development was not a significant predictor of the number of installs. Research is needed to identify other factors that may influence the number of installs, as that can help mHealth app development.

## Introduction

### Background

Mental health is integral to our salubrity, as reflected in the definition of health by the World Health Organization (WHO): “Health is a state of complete physical, mental and social well-being and not merely the absence of disease or infirmity” [1]. Mental disorders are very common, and studies have estimated that the cumulative global impact of mental disorders in terms of lost economic output amounted to US $16.3 billion between 2011 and 2030 [2]. Therefore, it is critical to provide accessible, timely, and inexpensive mental care [2], and this can be done with the help of information technology, such as mobile health (mHealth), “the delivery of healthcare services via mobile communication devices” [3].

An increasingly popular expression of mHealth is through smartphone apps: the global mHealth apps market, which is dominated by Apple App Store and Google Play Store, was valued at approximately US $8.0 billion in 2018 and is expected to have a compound annual growth rate of around 38.3% between 2019 and 2025, generating US $111.1 billion by 2025 [4]. To use an app from these stores, you have to install it, which requires downloading it and then running it on your device. However, there is no publicly available information in the Apple iOS App Store about the number of downloads of each app. Therefore, we focused our study on the apps available on the Google Play Store. Although the exact number of installs is not publicly available, each app in the Google Play Store is classified with a level of installs (described in detail in the Methods section), ranging from level 0 (ie, no installs) to level 19 (ie, between 1,000,000,000 and 5,000,000,000 installs).

Studies in economics (for generic, not health care–related apps) have identified several factors that positively affect the number of app installs, including lower price, higher number of user reviews and rating, and availability in both platforms (ie, Apple App Store and Google Play Store) [5,6]. A previous study in mHealth observed that cheaper apps with in-app purchases and higher user ratings and number of written reviews are more likely to have more downloads [7]. Furthermore, in a study of mHealth in urology, the participation of health care professionals (HCPs) in app development enhanced the apps’ probability of having a greater number of installs [7]. Other factors that have been associated with the number of app downloads are app size, the textual and visual description (ie, screenshots) of the app in the online store, updates, and age-restricted content [8-12].

### Objectives

Successful mHealth clinical implementations have been demonstrated in several mental conditions, such as anxiety, bipolar disease, depression, posttraumatic stress disorder, and schizophrenia [13-17]. However, although mental health apps can be used for self-monitoring, counseling, clinical practice support, and telemedicine, there are varying levels of adoption by users, as demonstrated by the discrepancy in the number of app installs in published articles [18-21]. However, to our knowledge, the factors that influence the number of installs of psychiatry apps have not been analyzed. Therefore, we aimed to identify predictors of the number of installs in psychiatry apps.

## Methods

### Study Outline and Research Procedure

**Figure 1:**
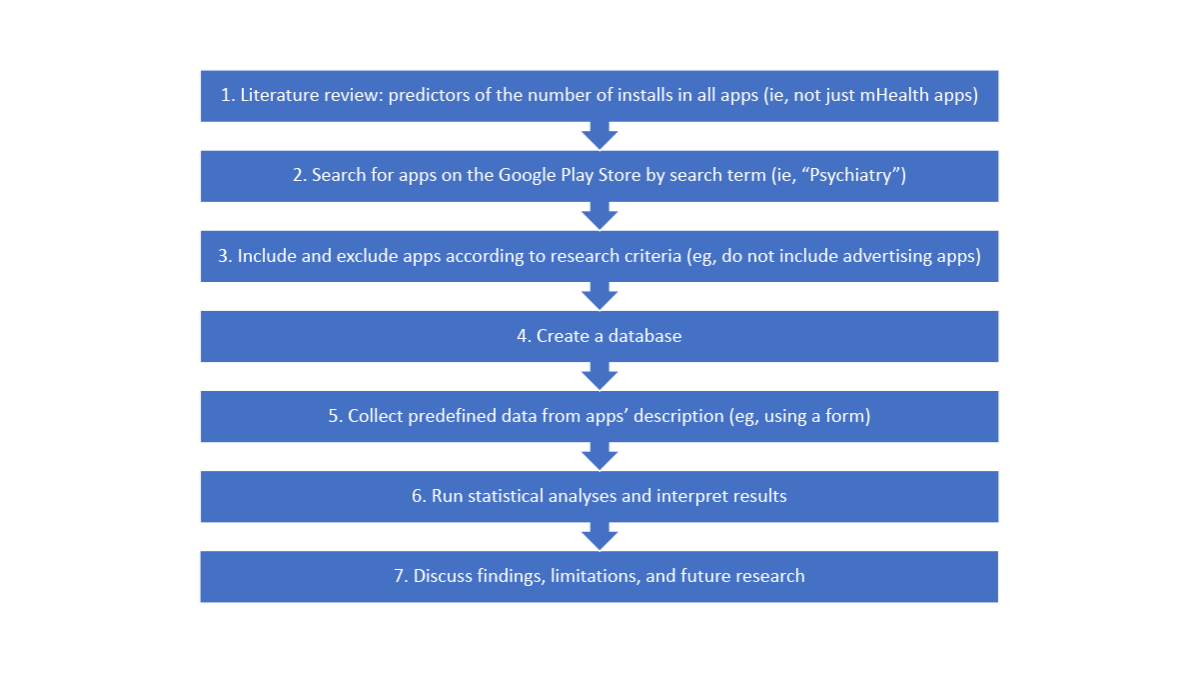
Study outline and research procedure. mHealth: mobile health.

A flow diagram with the process used in this study is represented in Figure 1.

### Search Strategy

A literature search using the search terms “Psychiatry,” “smartphone,” “tablet,” “Android,” “application,” “app,” “mHealth,” “installs,” “level,” “downloads,” “success,” “predictors,” “factors,” “determinants,” and “demand” was conducted using the PubMed, Google Scholar, Scopus, and Web of Science databases to find all the literature related to mHealth and psychiatry apps’ downloads up to May 1, 2019. Subsequently, the bibliography of the included articles was reviewed with the aim of locating relevant studies.

Simultaneously, a review of available psychiatry apps in the Google Play Store was conducted: all apps retrieved with the search term “Psychiatry” in their metadata (ie, the title, description, keywords, or version history) were examined. As some predictors (eg, app rating) or the dependent variable (ie, number of installs) might change, we decided to capture all the available Google Play Store data in a single day (April 9, 2019) as a snapshot. Only psychiatry-specific apps were included in this study; consequently, generic apps (ie, with content directed at several specialties, eg, a physiology book), ludic games (ie, nontherapeutical), and advertising apps (ie, related to a pharmaceutical product or a private office) were excluded.

Although the exact number of downloads is not explicit on Google Play Store, each individual app has a *level of installs*. Google Play Store publishes the amount of downloads an app has in incremental brackets: 0 (ie, no installs), 1 to 5, 5 to 10, 10 to 50, 50 to 100, 100 to 500, 500 to 1000, 1000 to 5000, 5000 to 10,000, 10,000 to 50,000, 50,000 to 100,000, 100,000 to 500,000, 500,000 to 1,000,000, 1,000,000 to 5,000,000, 5,000,000 to 10,000,000, 10,000,000 to 50,000,000, 50,000,000 to 100,000,000, 100,000,000 to 500,000,000, 500,000,000 to 1,000,000,000, and 1,000,000,000 to 5,000,000,000 installs. As there is no public information regarding the number of app installs for each individual app in the Apple App Store, this study only included apps available on Google Play Store.

### Predictor Variables for the Number of Installs

On the basis of previous economic studies of app demand that determined which factors influence *generic* (ie, not health care specific) app installs, 2 reviewers (MP and NA) recorded all available information for each app according to 14 predetermined variables: (1) number of installs, the dependent variable; (2) number of written user reviews; (3) price in US dollars; (4) average user rating (number of stars from 1 to 5); (5) app size (in megabytes); (6) number of screenshots (ie, an actual app image that showcases its features and functionality); (7) length of app description (number of characters in the app description not including spaces); (8) app availability in the Apple App Store (ie, whether the app is also available for iOS smartphones or tablets); (9) new versions available (ie, whether the app has been updated since launch); (10) absence of age restriction (ie, defined by the developer as having content appropriate for all ages); (11) availability of in-app purchases (ie, the opportunity to buy extra content); (12) participation of a psychiatrist (ie, psychiatrist or psychiatry association); (13) participation of another HCP (ie, other medical doctors, pharmacists, or nurses); (14) no HCP (ie, no explicit mention of an HCP). The identification of HCP participation was based on an examination of the app description or its website and was only considered to be present when explicitly mentioned. These variables are listed in Table 1. The list of predictors is presented as a form in Multimedia Appendix 1. Apps were not downloaded.

The 2 reviewers gathered data about the level of installs based on the classification system used by Google in the Play Store (Table 1). At the time of review (April 9, 2019), no psychiatry app had been installed over 50,000,000 times.

### Statistical Analyses

Analyses were performed using SPSS v20 (IBM Statistics). *P*<.05 was considered statistically significant in all analyses. Descriptive analyses were conducted, and a multivariate ordinal logistic regression analysis was performed to identify the factors predicting the number of installs for each app.

**Table 1:** The variables included in the model and their annotations.

Variables		Description
**Level of installs^a^**		
	Level 0	no installs
	Level 1	1-5 installs
	Level 2	6-10 installs
	Level 3	11-50 installs
	Level 4	51-100 installs
	Level 5	101-500 installs
	Level 6	501-1000 installs
	Level 7	1001-5000 installs
	Level 8	5001-10,000 installs
	Level 9	10,001-50,000 installs
	Level 10	50,001-100,000 installs
	Level 11	100,001-500,000 installs
	Level 12	500,001-1,000,000 installs
	Level 13	1,000,001-5,000,000 installs
	Level 14	5,000,001-10,000,000 installs
	Level 15	10,000,001-50,000,000 installs
Number of reviews		Number of reviews in the Google Play Store
Actual price		Actual price of the app in US dollars
Average user rating		User evaluation on a scale from 1 to 5 stars
App size		App file size in megabytes
**No age restriction^b^**		
	0	Age restriction
	1	No age restriction (ie, appropriate for all ages)
Number of screenshots		Number of screenshots in the Google Play Store
Length of description		Number of characters (without spaces) in the textual app description in the Google Play Store
**Availability in the Apple App Store^c^**		
	0	Not available
	1	Available
**Version**		
	0	One version
	1	New version exists
**In-app purchases**		
	0	No in-app purchase
	1	In-app purchase available
**Psychiatrist participation**		
	0	Other
	1	Psychiatrist- or psychiatry association
**HCP^d^** **participation other than psychiatrist**		
	0	Other
	1	Other HCPs, pharmacists, and nurses
**No HCP participation**		
	0	Other
	1	No HCP mentioned

^a^The exact number of installs is not available from the Google Play Store. We categorized it according to the system used by Google in the Play Store.

^b^Apps without age-restricted content.

^c^Available on the Apple App Store.

^d^HCP: health care professional.

## Results

### Descriptive Statistics

The PubMed search identified 1 study on the predictors of downloads in mHealth smartphone apps, but it did not reveal any studies on the predictors of the number of installs for psychiatry apps, suggesting that this is the first study of its kind. However, studies in economics were found on Google Scholar, which determined the predictors of downloads for generic apps and were tested in this study.

We performed a search on the Google Play Store on the April 9, 2019. A total of 250 Android apps contained the term “Psychiatry” in their metadata. Among them, 122 apps were excluded: 119 were generic apps (ie, not designed specifically for psychiatry, eg, “Medicine: diagnosis, clinical cases, Tumor Node Metastasis, International Classification of Diseases”) and 3 were just for making appointments or advertisement (eg, “Shantvan Clinic”).

Of the 128 included apps (Multimedia Appendix 2), 72.7% (93/128) were free. Of the paid apps, the prices ranged from US $2.99 (several apps) to US $209.99 (“Principles and Practice of Geriatric Psychiatry 3”), with a median price of US $26.39. The average app rating was less than 3 stars (average 2.76), and 83.6% (107/128) apps had no written review at the time of the study. On average, each app had 8.87 screenshots, and the length of the description varied from 75 to 3456 characters (without spaces; Table 2).

**Table 2:** Summary of descriptive statistics for continuous variables.

Variables		Mean (SD)	Range	Median
Number of reviews		2739.05 (27,958.02)	0-314,639.00	0
**Actual price in US dollars**				
	All apps	11.04 (29.22)	0-209.99	0
	Paid apps	40.39 (44.37)	2.99-209.99	26.39
Average user rating		2.76 (2.07)	0-5	3.85
App size		11.96 (18.03)	0.29-141	6.55
Number of screenshots		8.87 (5.73)	2-24	8
Length of description		1253.23 (822.42)	75-3456	1301.50

**Figure 2:**
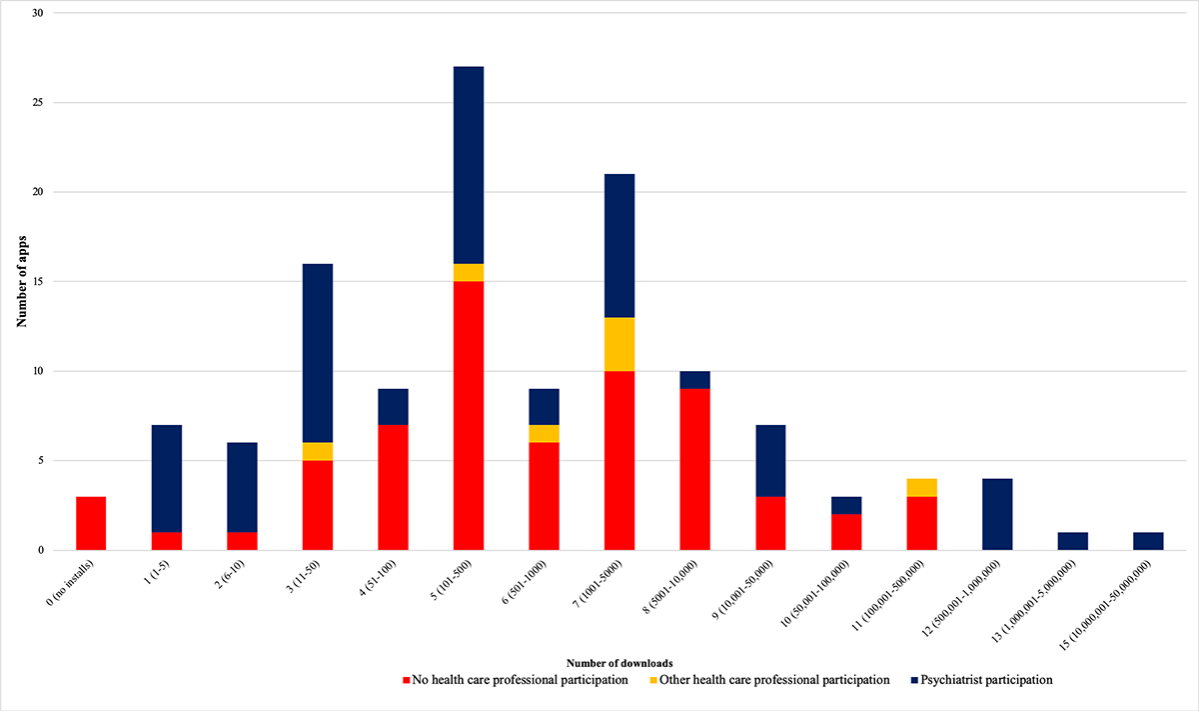
Number of apps per level of installs and health care professional participation.

Figure 2 shows the number of apps in each level of installs and HCP participation (ie, psychiatrists or psychiatry association, other HCP, or no HCP at all). There was a wide variation in HCP participation in each level of downloads, ranging from 0% (0/3) in apps without any download to 100% (6/6) in all apps with more than 500,000 installs, which included levels 12 (ie, between 500,001 and 1,000,000 installs), 13 (ie, between 1,000,001 and 5,000,000), and 15 (ie, between 10,000,001 and 50,000,000 installs).

Although 0.8% (1/128) of the apps had between 10,000,000 and 50,000,000 installs, approximately half 53.1% (68/128) had less than 500 and 2.3% (3/128) had never been installed (Table 3).

Less than half of the apps 43.8% (56/128) were developed with psychiatrists’ input, and other HCPs were involved in development of 5.5% (7/128) of the apps; 50.8% (65/128) of the apps had no documented HCP involvement. Furthermore, 95.3% (122/128) of the apps had no age restriction. Only 21.9% (28/128) had in-app purchases (Table 3).

**Table 3:** Frequencies for the categorical and binary variables (N=128).

Variables		Frequency (%)	Cumulative percentage (%)
**Level of installs**			
	Level 0: no installs	3 (2.3)	2.3
	Level 1: 1-5 installs	7 (5.5)	7.8
	Level 2: 6-10 installs	6 (4.7)	12.5
	Level 3: 11-50 installs	16 (12.5)	25.0
	Level 4: 51-100 install	9 (7.0)	32.0
	Level 5: 101-500 installs	27 (21.1)	53.1
	Level 6: 501-1000 installs	9 (7.0)	60.2
	Level 7: 1001-5000 installs	21 (16.4)	76.6
	Level 8: 5001-10,000 installs	10 (7.8)	84.4
	Level 9: 10,001-50,000 installs	7 (5.5)	89.8
	Level 10: 50,001-100,000 installs	3 (2.3)	92.2
	Level 11: 100,001-500,000 installs	4 (3.1)	95.3
	Level 12: 500,001-1,000,000 installs	4 (3.1)	98.4
	Level 13: 1,000,001-5,000,000 installs	1 (0.8)	99.2
	Level 14: 5,000,001-10,000,000 installs	0 (0)	99.2
	Level 15: 10,000,001-50,000,000 installs	1 (0.8)	100.0
**No age restriction**			
	Age restriction	6 (4.7)	4.7
	No age restriction	122 (95.3)	100.0
**Availability in Apple App Store**			
	Not available	83 (64.8)	64.8
	Available	45 (35.2)	100.0
**Version**			
	Only 1 version	29 (22.7)	22.7
	More than 1 version exists	99 (77.3)	100.0
**In-app purchases**			
	No in-app purchase	100 (78.1)	78.1
	In-app purchase available	28 (21.9)	100.0
**Psychiatrist** **participation**			
	Other	72 (56.3)	56.3
	Psychiatrist- or psychiatry association	56 (43.8)	100.0
**Other HCP^a^** **participation**			
	Other	121 (94.5)	94.5
	Other HCPs, pharmacists, and nurses	7 (5.5)	100.0
**No HCP** **participation**			
	Other	63 (49.2)	49.2
	No HCP mentioned	65 (50.8)	100.0

^a^HCP: health care professional.

### A Multivariate Logistic Regression Analysis

A multivariate logistic regression analysis revealed the factors that influence the number of installs among psychiatry apps (Table 4).

Cheaper apps (*P*<.001), apps with higher user rating (*P*<.001), and apps with available in-app purchases (*P*<.001) were significantly associated with app installs. Moreover, apps with age restriction were more likely to have a greater number of installs than apps without age restriction.

Although only apps with HCP participation had more than 500,000 installs (ie, levels 12-15), the explicit involvement of a psychiatrist or another HCP in the development of the app was not statistically significantly associated with the number of app installs. All other evaluated factors (ie, number of reviews, app size, number of screenshots, length of description, availability in the Apple App Store, and new published versions) were also not statistically significant predictors.

The Nagelkerke *R*^2^ statistic, which measures the strength of the association between the dependent variable and the predictor variables, was moderate.

**Table 4:** Multivariate ordinal logistic regression analysis.

Variables^a,b^	Estimates^c^	SE	*P* value	95% CI
Other health care professionals’ participation	−0.186	0.741	.80	−1.64 to 1.27
Psychiatrist participation	−0.583	0.451	.19	1.47 to 0.3
Number of reviews	0.000089	0.000059	.13	−0.000027 to 0.0002
Actual price in US dollars	−0.031	0.008	<.001	−0.05 to −0.01
Average user rating	0.631	0.099	<.001	0.44 to 0.83
App size	−0.002	0.013	.86	0.03 to 0.02
No age restriction	−1.722	0.829	.04	3.35 to −0.097
Number of screenshots	0.014	0.031	.64	0.05 to 0.08
Length of description	0.0001	0.0002	.63	−0.0003 to 0.001
Availability in the Apple App Store	0.517	0.417	.22	−0.3 to 1.33
Version	0.536	0.425	.21	−0.297 to 1.37
In-app purchases	1.67	0.459	<.001	0.77 to 2.57
Nagelkerke *R*^2^	—^d^	—	.62	—

^a^The dependent variable is the level of installs.

^b^The reference level for HCP participation is *No HCP participation*.

^c^Estimates are the ordered log-odds regression coefficients, and they show the relative magnitude (ie, relative impact of the factor) and direction (ie, positive or negative) of impact of the listed variables on the level of installs.

^d^Not applicable.

## Discussion

### Principal Findings

This study is innovative in psychiatry because it shows that a lower price, optional in-app purchases, age restriction, and a higher rating positively influence the number of app installs. This is in line with studies in economics that identified predictors of the number of downloads in non-mHealth apps: lower price, available in-app purchases, smaller app size, more textual and visual descriptions, and version updates [5,6,8-12].

Although a lower price has been identified as a significant predictor of downloads, and it has been shown that the possibility of in-app purchases can positively affect a user’s decision to download the app, some users opt to pay upfront for a more complete app, whereas others only download free apps, even if they have limited features [6].

Online word of mouth (ie, online exchange of opinions) influences ecommerce sales, including smartphone apps [9]. Online word of mouth has 2 main features: volume (the amount of word of mouth that generates the cognitive consequence of awareness) and valence (whether it is positive or negative that produces the cognitive consequence of attitude). In commercial app stores (ie, Apple App Store and Google Play Store), user rating and reviews may be perceived as reflecting previous users’ experience: the number of reviews as the volume and the user rating as the valence, although it has been shown that there is no correlation between mental app ratings and the apps’ quality of information or adherence to best practice guidelines [6,10,14]. Moreover, because most mHealth apps are available on commercial stores, the decision to download an app can be influenced by the apps’ information (eg, title, description, developer, and screenshots). In addition, having the same app available on the 2 most popular mHealth app platforms (ie, Apple App Store and Google Play Store) may raise awareness about it, thereby influencing the number of downloads [6].

HCP participation can be a proxy of scientific integrity, and it has been hypothesized that establishing scientific evidence for commercial mHealth apps can promote their adoption in health care practice and improve clinical outcomes [22]. Moreover, a study in mHealth identified HCP participation as a significant predictor of download of urology apps [23]. However, in our Android Play Store psychiatry-specific sample, although an HCP was documented in all apps that had more than 500,000 installs, the explicit participation of psychiatrists in app development was not a significant predictor of the number of installs. Moreover, only half of the apps had explicit scientific expert input, and when HCP participation was mentioned, there was no objective method to measure the extent of that involvement or a guaranteed method to assess if it was actually true. Potentially, this can be resolved by requiring mHealth apps to have a detailed disclosure form (eg, similar to scientific publications) or by implementing an independent certification of HCP participation. This would be beneficial toward the functional certification and content regulation of mHealth apps.

As mHealth apps are becoming increasingly popular, for both professionals and patients, the lack of evidence to recommend a specific mental mHealth app in favor of another becomes a pressing issue, and several pitfalls have been identified, such as outdated information or misinformation, often created by lay people, with disregard for usability and scientific evidence [24-27]. Moreover, warnings have been issued because of subpar safety, inadequate privacy policies, questionable content, and even dangerous nature of mental health apps [28-30].

To address these problems, it has been suggested that HCPs should have a pivotal position in the development, review, and recommendation of mHealth apps [27]. This can either be done individually or through scientific societies, which could coordinate this effort. A pragmatic stance has been taken by the American Psychiatric Association (APA), which devised a step-by-step App Evaluation Model [31] in which psychiatrists are advised the following:

1. To begin by collecting background information on the app (eg, who is the developer and what is the business model)
2. To exclude risk, privacy, and security issues (eg, does the app have a privacy policy, which personal data are collected, and are the data available to any third party)
3. To evaluate the evidence (eg, is there peer-reviewed, published evidence about the app or the science behind it)
4. To evaluate how easy is it to use (ie, evaluate its usability)
5. Assess interoperability (ie, how easy is it to share the data in the app with other health care software).

The APA’s step-wise approach is built so that if, for example, there are privacy concerns, the app is considered dangerous and therefore excluded without having to evaluate other factors [31]. By taking an active role in mHealth, HCPs can safeguard the apps’ up-to-date scientific evidence and, concurrently, promote user safety and privacy. This is in line with the WHO’s Mental Health Action Plan 2013-2020: promote mental well-being; prevent mental disorders; provide care; enhance recovery; promote human rights; and reduce the mortality, morbidity, and disability in people with mental disorders [1].

### Limitations

This study has limitations, in addition to the impossibility of controlling all factors (either mathematically or not measurable) that may influence the number of installs in the real world. Our study sample was restricted to the Google Play Store because Apple does not publish an individual app’s number of downloads. Instead, Apple lists the *Top 200 Medical Apps*, ranked by a undisclosed proprietary algorithm, which prevents further analysis from being performed as there was no way of inferring the number of installs from the position within that list. Furthermore, at the time of the review, there were no psychiatry apps in that *Top 200 Medical Apps* list. Moreover, our search was performed in the US store, which may not be representative of other locations. Identifying psychiatry apps on Google Play Store is dependent on Google’s search algorithm and is not straightforward. Therefore, to avoid entropy, we decided to perform a search using just the term “Psychiatry.” Future research might determine the predictors of the number of installs for specific mental health keywords (eg, “anxiety,” “depression,” or “schizophrenia”).

### Conclusions

Mental health apps can be used by patients and HCPs in a myriad of contexts, from academic research to clinical practice. Our study shows that psychiatry apps with a lower price, optional in-app purchases, age restriction, and a higher rating are expected to have a higher number of installs. Research is needed to identify other factors that may influence the number of installs, as that can help mHealth app development.

## Appendix

Multimedia Appendix 1 List of predictors.

Multimedia Appendix 2 List of included apps.

## Abbreviations

APA: American Psychiatric Association
HCP: health care professional
mHealth: mobile health
WHO: World Health Organization
