# Supplementary material for: Predictors of the Number of Installs in Psychiatry Smartphone Apps: Systematic Search on App Stores and Content Analysis
Source: JMIR Ment Health. 2019 Nov 19;6(11):e15064. doi: 10.2196/15064 (PMC6891398; doi:10.2196/15064)
Supplement: Multimedia Appendix 2 [file mental_v6i11e15064_app2.pdf]

App name	Level of Installs	HCP
2nd Congress on Evidence based Psychiatry	11-50	HCP_Psychiatry
AACAP	1001-5000	HCP_Psychiatry
ACLP Events	101-500	HCP_Psychiatry
AJGP	501-1000	HCP_Psychiatry
All Mental Disorders and Treatment	10,001-50,000	No HCP
American Psychiatric Association Meetings	1001-5000	HCP_Psychiatry
APA eBooks	101-500	HCP_Psychiatry
APA Meetings	1001-5000	HCP_Psychiatry
APA Style Citing & Referencing Guide	1001-5000	No HCP
Basic Psychology Book To Learn Basic Psychology	5001-10,000	No HCP
Become Psychiatrist	501-1000	No HCP
Biblioclick in Psychiatry	501-1000	No HCP
Biological Psychiatry	1001-5000	HCP_Psychiatry
Biological Psychiatry Congress 2017	101-500	HCP_Psychiatry
Bipolar Test	1001-5000	No HCP
BSDR Player	1001-5000	No HCP
Child and Adolescent Psychiatr	1-5	HCP_Psychiatry
Child and Adolescent Psychiatry Exam Prep 2019	1-5	No HCP
Child and Adolescent Psychiatry Exam Prep 2019 free	51-100	No HCP
Clinical Psychiatry Medicine	501-1000	No HCP
CURRENT Diagnosis & Treatment Psychiatry	11-50	HCP_Psychiatry
CURRENT Diagnosis &Treatm Psy	11-50	HCP_Psychiatry
DBT Daily	5001-10,000	No HCP
Drugs in Psychiatry, 2nd Ed	11-50	HCP_Psychiatry
DSM 5 Practice Exam Prep 2019	10,001-50,000	No HCP
DSM-5 Diagnostic Criteria	1001-5000	HCP_Psychiatry
DSM-5 Differential Diagnosis	10,001-50,000	HCP_Psychiatry
DSM-V CIE-11.	5001-10,000	HCP_Psychiatry
Easy Psychiatry	501-1000	No HCP
Electroconvulsive Therapy-ECT	501-1000	HCP_Psychiatry

Emergency Psychiaytry Exam Prep Flashcards & Notes	no installs	No HCP
EPA 2019	101-500	HCP_Psychiatry
Eureka: Psychiatry	1-5	HCP_Psychiatry
Fight Depression Naturally	50,001-100,000	HCP_Psychiatry
How to Become a Psychiatrist	51-100	No HCP
IACAPAP Text	101-500	HCP_Psychiatry
Indian J Psychiatry	1001-5000	HCP_Psychiatry
Indian Journal of Psychiatry	11-50	HCP_Psychiatry
INPP 2016	101-500	HCP_Psychiatry
International Journal of Psychology	1001-5000	Other HCP
JAACAP	1001-5000	HCP_Psychiatry
Maudsley Prescribing Guidelines in Psychiatry 12th	11-50	HCP_Psychiatry
Maudsley Prescribing Guidelines in Psychiatry 13th	11-50	HCP_Psychiatry
Mental disorders	50,001-100,000	No HCP
Mental disorders	100,001-500,000	No HCP
Mental Disorders	100,001-500,000	No HCP
Mental Disorders and Treatment	101-500	No HCP
Mental Health	101-500	No HCP
Mental Health Book Apps	1001-5000	Other HCP
Mental Health Nursing	10,001-50,000	HCP_Psychiatry
Mental Health Quotes	1001-5000	No HCP
Mental Health Tests	50,001-100,000	No HCP
MGH Psychiatry	51-100	HCP_Psychiatry
Moodpath - Depression & Anxiety Test	500,001-1,000,000	HCP_Psychiatry
Mount Sinai Expert: Psychiatry	6-10	HCP_Psychiatry
MRCPsych Learn A	no installs	No HCP
Neurology & Psychiatry – Dict	10,001-50,000	HCP_Psychiatry
NOCD: OCD Treatment App	1001-5000	HCP_Psychiatry
Nursing Psychiatry	11-50	No HCP
Nursing Psychiatry Deluxe	51-100	No HCP
Obsessive-Compulsive & Rela 2e	1-5	HCP_Psychiatry
Oxford American H. Psychiatry	11-50	HCP_Psychiatry
Oxford Asses & Progress:	6-10	HCP_Psychiatry

Psych		
Oxford Handbook Psychiatry, 3e	51-100	HCP_Psychiatry
PA Psychiatry QA Review	51-100	No HCP
Pacifica - Stress & Anxiety	1,000,001-5,000,000	HCP_Psychiatry
Peak – Brain Games & Training	10,000,001-50,000,000	HCP_Psychiatry
Personal Zen	501-1000	Other HCP
Personality Disorder	6-10	HCP_Psychiatry
PMS – Psychiatric Mnemonic Series	101-500	No HCP
Prescriber's Guide, Stahl's Psychopharmacology, 6e	11-50	HCP_Psychiatry
Prescrições em Psiquiatria	101-500	HCP_Psychiatry
Princi Geriatric Psychiatry, 3	1-5	HCP_Psychiatry
Psy Tests	500,001-1,000,000	HCP_Psychiatry
PsyCare - mental wellness and healthcare	101-500	HCP_Psychiatry
Psych Meds	10,001-50,000	No HCP
Psych Meds Lab Guide	1001-5000	No HCP
Psychiatric & Mental Health Nurse Exam Prep	6-10	No HCP
Psychiatric & Mental Health Nurse Exam Prep 2019 free	101-500	No HCP
Psychiatric and Mental Health Nursing Quiz	101-500	No HCP
Psychiatric Exam Prep	1001-5000	No HCP
Psychiatric Mnemonics	1001-5000	No HCP
Psychiatric Nursing 2019	11-50	Other HCP
Psychiatric Nursing Exam Prep	5001-10,000	No HCP
Psychiatric Nursing Notes	5001-10,000	No HCP
Psychiatric Nursing Quiz	5001-10,000	No HCP
Psychiatry	101-500	No HCP
Psychiatry	501-1000	No HCP
Psychiatry & Psychology	1001-5000	No HCP
Psychiatry 101	101-500	No HCP
Psychiatry Addiction	101-500	No HCP
Psychiatry by Dr. Praveen	10,001-50,000	HCP_Psychiatry
Psychiatry Child Adolescent	101-500	No HCP
Psychiatry EXAM 2018 Ed	101-500	No HCP
Psychiatry Exam Prep 2018	1001-5000	No HCP
Psychiatry Exam Questions 2018	5001-10,000	No HCP
Psychiatry Exam	51-100	No HCP

StudyToken		
Psychiatry Flashcards	1001-5000	No HCP
Psychiatry Flashcards 2018 Ed	51-100	No HCP
Psychiatry Flashcards Plus	51-100	No HCP
Psychiatry Flashcards Premium	11-50	No HCP
Psychiatry Flashcards Ultimate	11-50	No HCP
Psychiatry Geriatric	101-500	No HCP
Psychiatry Hub for PGs & USMLE	5001-10,000	No HCP
Psychiatry LANGE Q&A	5001-10,000	No HCP
Psychiatry Pocket	1001-5000	Other HCP
Psychiatry Pro-Diagnosis,Treatment & Psychotherapy	11-50	No HCP
Psychiatry QA Review	101-500	No HCP
Psychiatry Shelf QA Review	101-500	No HCP
Psychiatry Test Prep 2018 Ed	501-1000	No HCP
Psychiatry Test Prep PRO 2018 Ed	no installs	No HCP
Psychiatry USMLE Flash Cards 2018 Ed	101-500	No HCP
Psychiatry USMLE Quiz 2018 Ed	101-500	No HCP
PsychiatryUK	101-500	HCP_Psychiatry
PsychNotes: Clinical Pocket Guide	101-500	HCP_Psychiatry
PsychQbase	11-50	No HCP
Rapid Psychiatry, 2nd Edition	11-50	HCP_Psychiatry
Remente - Self Improvement	500,001-1,000,000	HCP_Psychiatry
Schizophrenia (Oxford Psychiatry Library), 2ed	1-5	HCP_Psychiatry
Sports Psychiatry	1-5	HCP_Psychiatry
Switch Tables	101-500	Other HCP
Synops Pscyhia Behav Clinic 11	6-10	HCP_Psychiatry
TAG - Threshold Assessment Grid	101-500	HCP_Psychiatry
The Maudsley Handbook Practi 6	6-10	HCP_Psychiatry
Treatment of Mental illness arabo	5001-10000	No HCP
Welltory: EKG heart rate	100,001-500,000	Other HCP

monitor and stress relief		
What's Up? - Mental Health App	100,001-500,000	No HCP
Youper	500,001-1,000,000	HCP_Psychiatry
